# Exploring the impacts of ketogenic diet on reversible hepatic steatosis: initial analysis in male mice

**DOI:** 10.3389/fnut.2024.1290540

**Published:** 2024-03-21

**Authors:** Gaetan Ravaut, Anthony Carneiro, Catherine Mounier

**Affiliations:** CERMO-FC Research Center, Molecular Metabolism of Lipids Laboratory, Biological Sciences Department, University of Quebec in Montreal (UQAM), Montreal, QC, Canada

**Keywords:** ketogenic diet, hepatic steatosis, inflammation, glucose intolerance, insulin resistance, rescue

## Abstract

Metabolic dysfunction-associated fatty liver disease (MAFLD) is the most common chronic liver disease. Ketogenic diet (KD), a diet with very low intake in carbohydrates, gained popularity as a weight-loss approach. However, in mice models, it has been reported that an excess exposition of dietary fat induces hepatic insulin resistance and steatosis. However, data published is inconsistent. Herein, we investigated in a mouse model, the metabolic effects of KD and its contribution to the pathogenesis of NALFD. Mice were exposed to KD or CHOW diet for 12 weeks while a third group was exposed to KD for also 12 weeks and then switched to CHOW diet for 4 weeks to determine if we can rescue the phenotype. We evaluated the effects of diet treatments on fat distribution, glucose, and insulin homeostasis as well as hepatic steatosis. Mice fed with KD developed glucose intolerance but not insulin resistance accompanied by an increase of inflammation. KD-fed mice showed an increase of fat accumulation in white adipose tissue and liver. This effect could be explained by an increase in fat uptake by the liver with no changes of catabolism leading to MAFLD. Interestingly, we were able to rescue the phenotype by switching KD-fed mice for 4 weeks on a CHOW diet. Our studies demonstrate that even if mice develop hepatic steatosis and glucose intolerance after 12 weeks of KD, they do not develop insulin resistance and more importantly, the phenotype can be reversed by switching the mice from a KD to a CHOW.

## 1 Introduction

Metabolic disorders (MD) are a group of conditions that affect metabolism homeostasis a central process for conversion of food into energy and for maintenance of tissue integrity. This syndrome is often associated with insulin resistance in response to elevated dietary fats (1). One of the most important contributors to metabolic syndrome (MS) is obesity. One approach to treat obesity and associated metabolic disorders are dietary interventions. Such as ketogenic diet (KD) (2, 3). This diet is typically very low in carbohydrates (less than 5% of daily calories) and very high in lipids (more than 80%) (4). For decades, the KD has been also used to treat epilepsy, especially in children with seizures (5, 6). This diet creates a metabolic state called ketosis, characterized by an increased level of plasmatic ketone bodies (7).

Due to the presence of a large proportion of fat in KD, it has been reported that this diet could participate in the development of hepatic steatosis also called metabolic dysfunction-associated fatty liver disease (MAFLD). MAFLD development is indeed highly correlated with a high dietary fat ingestion (8–10). This leads to the increase of fatty acid anabolism in liver driven by the activation of the master regulator of *de novo* lipogenesis and fatty acid uptake, sterol regulatory element-binding protein-1 (SREBP-1) (11). In humans, this ectopic accumulation of fat observed in MAFLD is also associated with hypertriglyceridemia, low levels of high-density lipoprotein (HDL) – cholesterol, glucose intolerance, insulin resistance leading to the development of type 2 diabetes (T2D) (12–14). Hyperglycemia and hyperlipidemia also disrupt the insulin signaling pathway activating stress and inflammation pathways (15). In this case, protein kinase B (Akt) and p70S6 Kinase (P70S6K) phosphorylation are decreased and circulating inflammatory cytokines increase developing a nonalcoholic steatohepatitis (NASH) (16–19). At this stage of the disease, symptoms can be reversible, but they become irreversible when cirrhosis appears (20).

Recently, Grandl et al. (21) showed that when compared to a high fat diet (HFD), mice fed with KD for 3 days, containing 90% kcal from fat and 0.3% kcal from carbohydrate, developed hepatic insulin resistance as well as induce glucose resistance (21). More recently, Long et al. (22) showed that a low carbohydrate, high fat diet (79.1% kcal form fat and 3.8% kcal from carbohydrate) induce hepatic fibrosis and NASH associated with high inflammation and severe hepatic steatosis (22). However, other studies showed opposite effects of KD on metabolic features. When comparing athletes fed a regular western diet to athletes fed with KD, the latter enhanced ketosis, demonstrating a decrease in body fat and an increase of lean mass. This is associated with a decrease in plasmatic inflammatory cytokines levels (such as IL-6, IL-1β, and TNFα) (23). Moreover, two human studies showed that obese patients, with or without T2D on KD for 12 weeks presented an amelioration of their metabolic profiles reflected by decreased plasmatic glycated-hemoglobinA1c, blood and hepatic triglycerides, hydrolysis by mitochondrion, low-density lipoprotein (LDL)-cholesterol concentration and fasting blood glucose and insulin levels while decreased (24, 25).

Ketogenic diets are used in a variety of cases, notably for their benefits in aging or epilepsy. In previous studies, several sources of fat were used, such as cocoa butter (26), vegetable oils (27) or lard (28, 29). Lard contained mainly saturated fat responsible for adverse metabolic effects (30). In mice, most of the studies have evaluated the effect of obesogenic diets by feeding the animals for 8 to 16 weeks, in order to observe significant metabolic changes (31–34). In addition, the ketogenic diets previously used contained various amount of carbohydrates (21–25). In the present study, we evaluated the effect of dietary saturated fats contained in lard and the total absence of carbohydrate in KD (84,5% fat from Lard, 0% carbohydrates) on mice’s metabolic profiles in order to evaluate the impact of KD as well as the absence of sugar. Animals were fed with the KD for 12 weeks and part of the mice were then switched on standard diet (CHOW) for an additional 4 weeks, general metabolic features were analyzed.

## 2 Materials and methods

### 2.1 Animals

C57BL/6 male mice were maintained at 23°C, 12 h dark-12 h light cycle. CHOW diet (D11112201) and ketogenic diet (KD, D16062902) were obtained from OpenSource Diet and ResearchDiet Inc., respectively. As summarized in the Supplementary Table S1, KD contains 5.5% fat (kcal) from soybean oil and 84.5 from lard. Carbohydrates represent less than 0.1%. 3 weeks old mice were fed *ad libitum* for 12 weeks with either CHOW diet or KD. Food intake was measured by weighing diet each day for CHOW and KD group and converted into calories. Part of the KD-fed mice were then switched to the regular CHOW diet for 4 more weeks (*n* = 3 per group). Mice were anesthetized in 2% isoflurane chamber and then euthanized in CO2 chamber at 12 weeks for two groups and at 17 weeks for the third group after 6 h fasting. Blood samples and liver tissues were collected. Liver tissues were fixed in 4% paraformaldehyde or flash frozen in liquid nitrogen. All experiments followed adherence to the care and use of laboratory animals. The animal protocol was approved by the Animal Care and Use Committee of the University of Quebec in Montreal.

### 2.2 Biochemical analysis

Plasmatic levels of insulin were determined using an Ultra-Sensitive Mouse Insulin ELISA Kit (CrystalChem) while plasmatic levels of ketone bodies were measured using the β-Hydroxybutyrate LiquiColor Assay (Stanbio). Liver triglycerides (TG) were determined using a triglyceride colorimetric assay kit (CaymanChemical). Glucose tolerance (GTT) and insulin tolerance (ITT) tests were assessed after 6 h diurnal fasting. Blood glucose concentration was measured from 2 μL of tail vein blood using an Accu-Check Aviva glucometer (Roche). For GTT, glucose level was evaluated at time: 0, 15, 30, 60, and 90 min after the intraperitoneal D-(+)-Glucose injection (1 g/kg body weight; Sigma-Aldrich, cat #G8270). For ITT, the glucose level was evaluated at time: 0, 15, 30, 45, and 60 min after the intraperitoneal injection of human recombinant insulin (0.5 U/kg body weight; Humulin R, Lilly).

### 2.3 Histology

Liver microtome sections (8 μm) were stained with RedOil and hematoxylin as previously described (35). Samples were visualized under white light using a Nikon Eclipse Ti microscope equipped with a Scion CFW-1612C color camera. Lipid droplets sizes and areas were quantified using the Particle Analysis function of the ImageJ software. The Particle Analysis function has been set to consider LD with a sphericity coefficient of 0.55–1.0 and a size range of 0.07–2,000 μm^2^.

### 2.4 Western blotting

Total proteins were extracted from liver samples as previously described (36). Denatured proteins were loaded on SDS-PAGE, and immunoblot analyses were carried out using the following primary antibodies: Akt (1:1000), P70S6K (1:1000), α-tubulin (1:1000), SREBP-1 (1:1000), Acetyl-CoA Coenzyme A (ACC) (1:1000), and Carnitine PalmytoylTransferase-1 (CPT1) (1:1000). Horseradish peroxidase (HRP)-conjugated anti-rabbit IgG (Abcam, cat. #ab6721) or anti-mouse IgG (Cell Signaling technology, cat. #7076) were used as secondary antibodies (1:1000). Bands intensities were quantified by the Analyze Gels function of the Image J software.

### 2.5 Quantitative retrotranscriptase-polymerase chain reaction

Total RNA was extracted from liver and adipose tissues using TRIzol Reagent (Life Technologies, cat. #15596-018; Carlsbad, United States) following the manufacturer’s instructions. One μg of RNA was reverse transcribed into cDNA using the ProtoScript II Reverse Transcriptase kit (New England Biolab). Quantitative PCR was performed on 50 ng cDNA using the following specific primers: Stearoyl-CoA desaturase 1 (SCD1) Fwd-5’-TCATACTGGTTCCCTCCTGC-3’/Rev-5’-TGCCTTGTAAGTTCTGTGGC-3’; Cluster of Differentiation 36 (CD36/FAT) Fwd-5’-GAATTAGAACCGGGCCACGTAGAAA-3’/Rev-5’-ACAGCTCCAGCAATGAGCCCAC-3’; Peroxisome Proliferator Activated Receptor alpha (PPARα) Fwd-5’-GGAAGACCACTCGCATTC-3’/Rev-5’-GTAATCAGCAACCATTGGGTCA-3′, Interferon beta (IFN-β) Fwd-5’-ATAGTCTCATTCCACCCAGTGC-3′/Rev-5’-AGAGTTACACTGCCTTTGCC-3′; Interleukin-4 (IL-4) Fwd-5’-GGAGATGGATGTGCCAAACG-3’/Rev-5’-CTTGGAAGCCCTACAGACGAG-3′ and Hypoxanthine Phosphoribosyl-Transferase (HPRT) Fwd-5’-TCAGTCAACGGGGGACATAAA-3/Rev-5’-GGGGCTGTACTGCTTAACCAG-3′. Reactions were performed using a Light Cycler 480 thermocycler (Roche, cat. #050152278001). Gene expression was normalized to Hprt1 expression and calculated using the comparative dCt method.

### 2.6 Statistical analysis

All data are presented as the mean +/− standard error of the mean. Independent replicas of each data point (*n*) are identified in figure legends. The Prism 9 software was used to performed Student’s *t*-tests or one way ANOVA when the test was appropriate. A one-tailed unpaired test was applied. A *p*-value <0.05 was regarded as statistically significant.

## 3 Results

### 3.1 Effect of KD on metabolic profile

**Figure 1:**
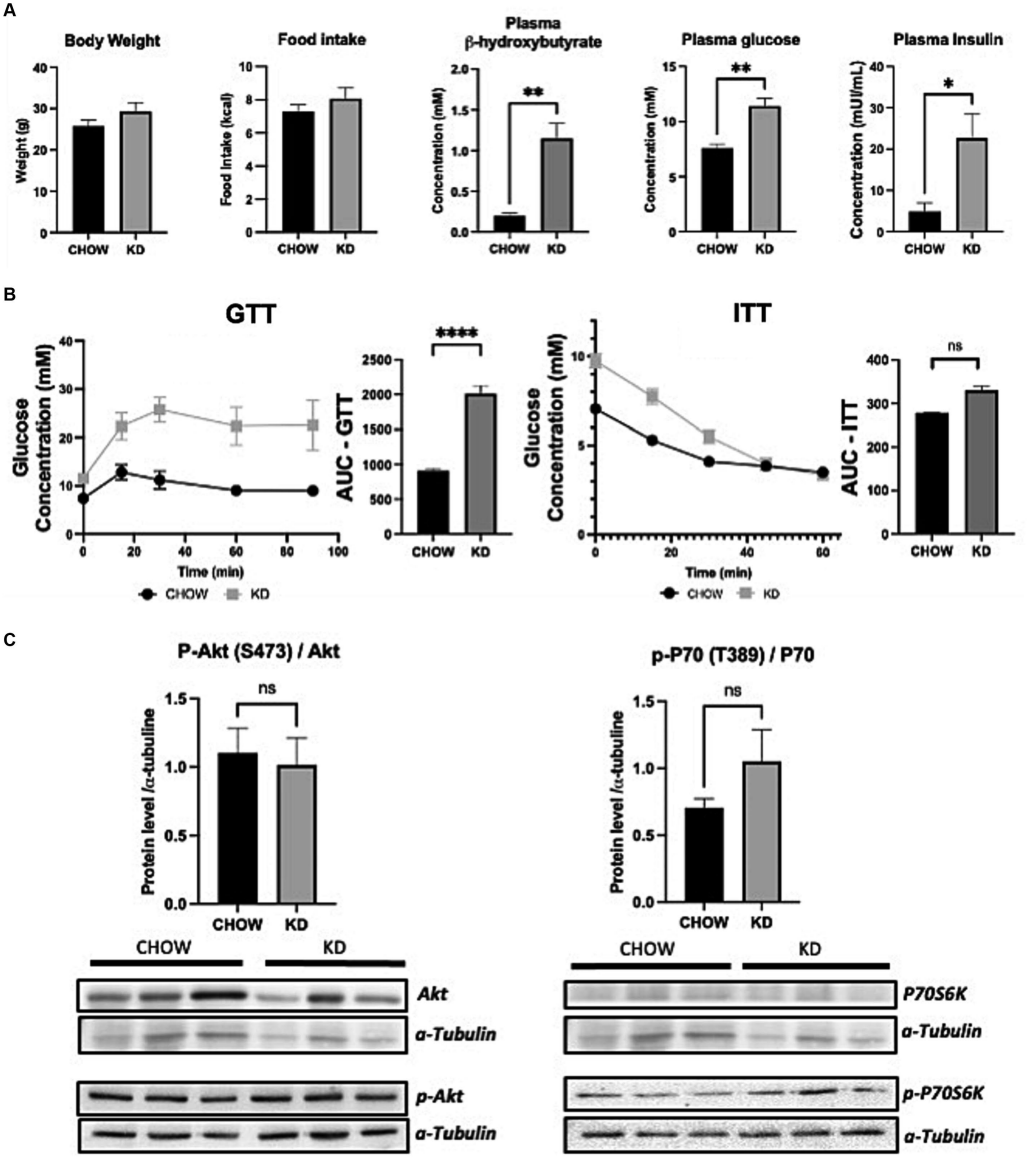
Metabolic profile and insulin sensitivity of mice fed *ad libitum* for 12 weeks with either CHOW or ketogenic diets (KD). **(A)** Evaluation of body weight, daily food intake plasma β-hydroxybutyrate and fasting glucose and insulin. **(B)** GTT (glucose tolerance test) was measured at week 9 and ITT (insulin tolerance test) was measured at week 10 after the beginning of the diet. AUC represents the calculated area under the curve. **(C)** Total and phosphorylated Akt and P70S6K levels measured in mice livers. Representative Western blots are depicted. α-tubulin was used as control (*n* = 3). Results are presented as mean +/− SEM (*n* = 3). ns, non-significant, *p-value <0.05, **p-value <0.01, and ****p-value <0.0001.

To evaluate the effect of KD on metabolic profile, we fed 3 weeks old C57BL/6 mice (just after weaning) with either standard diet (CHOW) or KD for 12 weeks. Mice consumed the same amount of calories and no change in body weight was observed between the two groups (Figure 1A). As expected, compared to chow diet, KD increased the plasmatic level of β-hydroxybutyrate (Figure 1A). KD-fed mice showed a higher fasting plasma glucose and insulin levels (Figure 1A), suggesting impairment in glucose homeostasis. However, KD-fed mice were still responsive to insulin as shown by ITT (Figure 1B), suggesting the absence of insulin resistance. To evaluate hepatic insulin resistance, we measured the phosphorylation state of AKT and P70S6K. As shown, in Figure 1C, the levels of phosphorylation of the two proteins, known to be impaired by insulin resistance, are similar between the two group of mice. Our data suggests that mice fed during 12 weeks with our KD, develop glucose intolerance but not insulin resistance.

### 3.2 Effect of KD on fat storage

**Figure 2:**
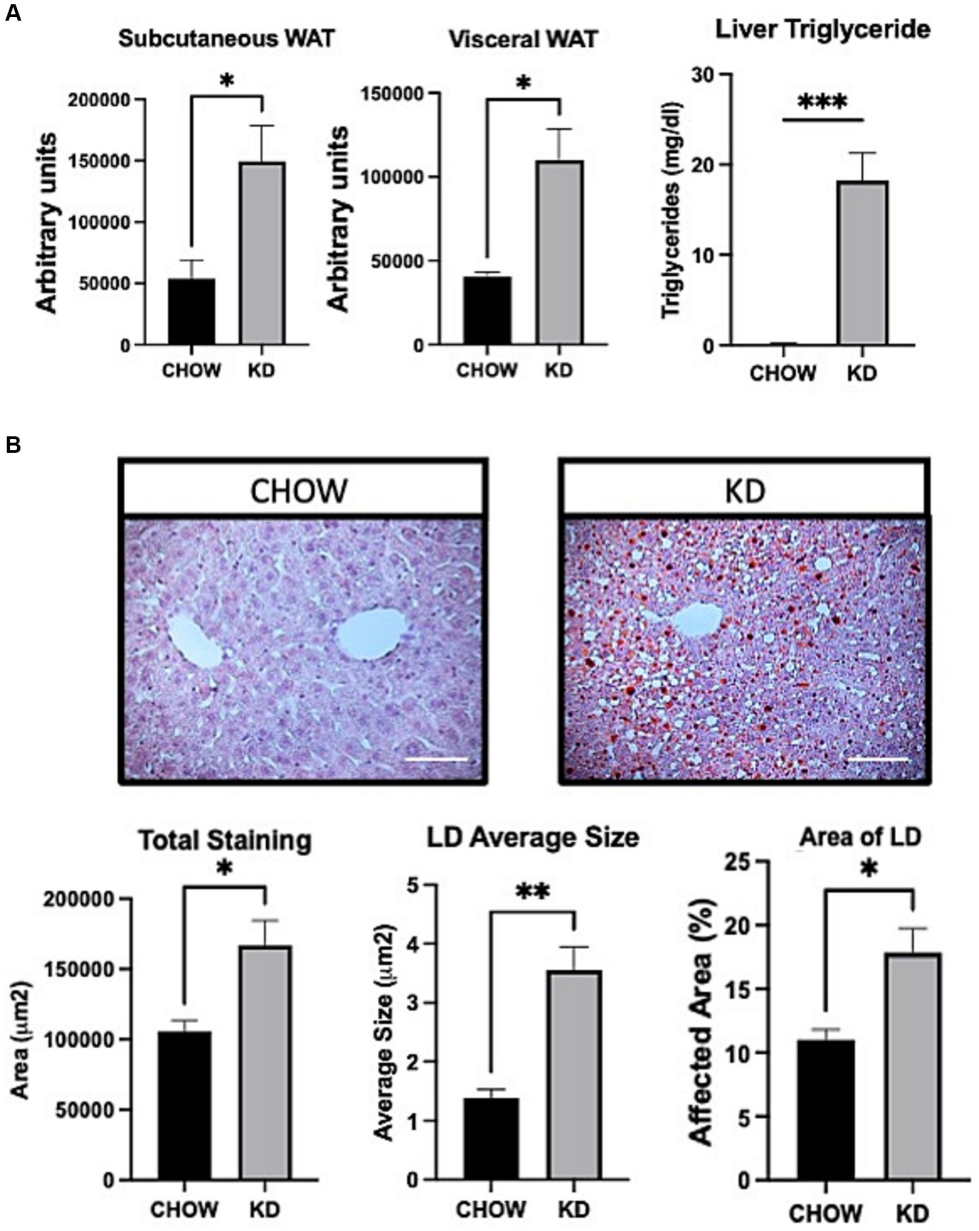
Adipose tissue and liver sections analysis in mice fed *ad libitum* for 12 weeks with either CHOW diet or Ketogenic Diet. **(A)** Representative images of white adipose tissue (WAT) distribution. Red arrows indicate subcutaneous WAT while blue arrows show visceral WAT. Scale Bar = 20 mm (Pictures in Supplementary Data). Quantifications were performed using the Particle Analysis function of ImageJ. Liver triglyceride levels. **(B)** Representative images of RedOil and eosin/hematoxyl staining of hepatic tissues sections. Scale bar = 70 μm. Quantification of total, average size area and percentage area of lipid droplets in liver sections were performed using ImageJ. Data are the mean +/− SEM. (*n* = 3). ns, non-significant, *p-value <0.05 and **p-value <0.01.

To evaluate the effect of KD on fat storage, we analyzed white adipose tissues and livers, in mice fed with either CHOW diet or KD. Compared to the CHOW-fed mice, KD-fed mice showed an increased mass of fat in both subcutaneous and visceral white adipose tissues (Figure 2A). In KD-fed mice compared to the control group, we observed a 3 folds increase in hepatic lipid droplets sizes. The LD area represented a higher percentage of total liver tissue and consequently, a larger affected total hepatic area (Figure 2B) demonstrating ectopic TG accumulation, the first step of hepatic steatosis development (Figure 2A). High consumption for 12 weeks of KD, mainly composed of saturated fat, leads to increase adiposity and hepatic steatosis.

### 3.3 Effect of KD on hepatic lipid metabolism

**Figure 3:**
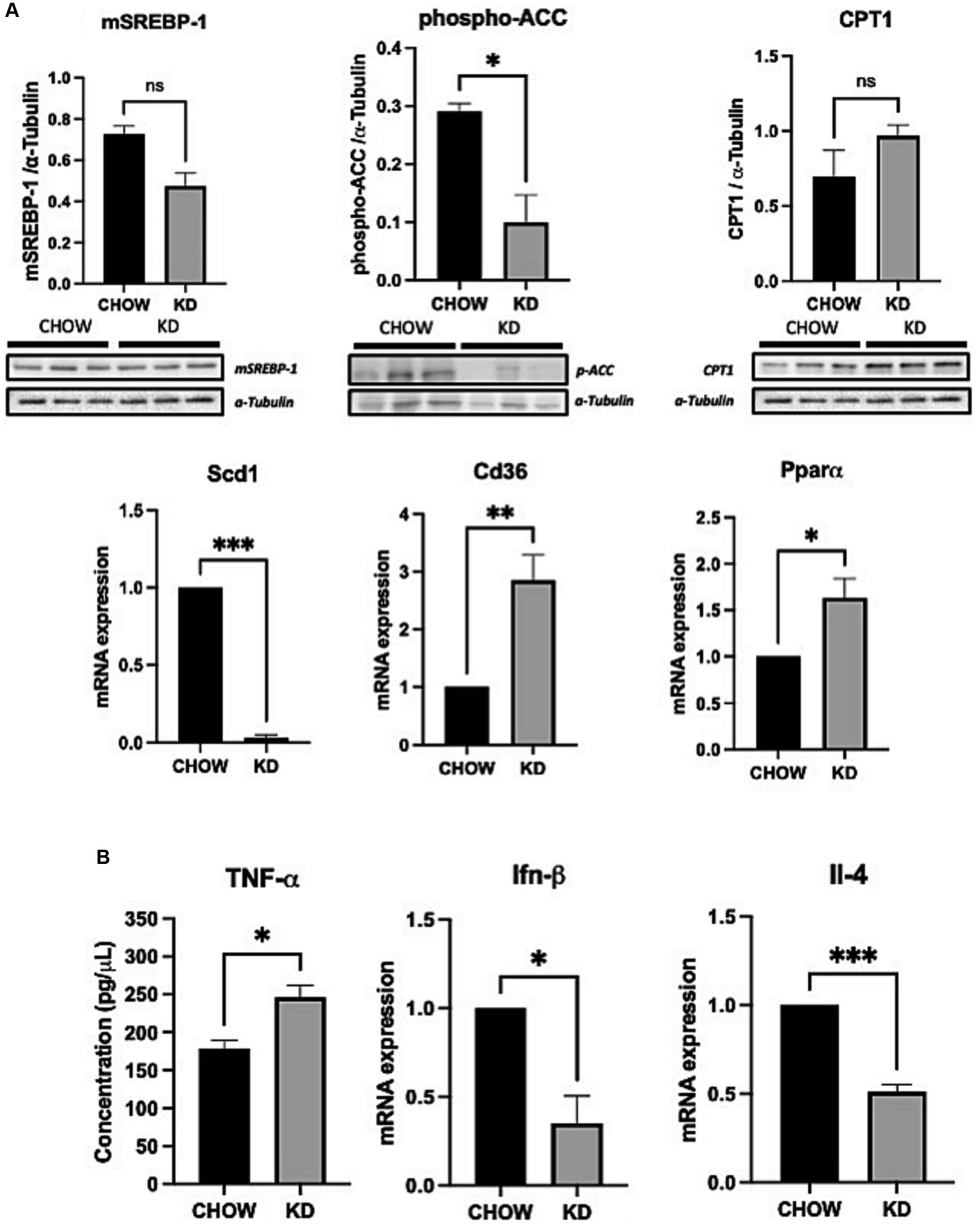
Hepatic expression of lipid metabolic genes and inflammatory cytokines in liver of mice fed *ad libitum* for 12 weeks with either CHOW diet or Ketogenic Diet. **(A)** Protein expressions of SREBP-1, phospho-ACC, CPT1 and mRNA expression of Scd1, Pparα and Cd36 are presented. α-TUBULINE and Hprt1 were used, respectively, as control. **(B)** Protein and mRNA expression of TNF-α (evaluated by ELISA) and Ifn-β and Il-4 are presented. Hprt1 was used as control for qPCR. Data are shown as mean +/− SEM. *n* = 3. Student’s *t*-test: *p-value <0.05, **p-value <0.01, ***p-value <0.001, and ****p-value <0.0001.

The development of hepatic steatosis often arises from an imbalance between lipid catabolism and lipid anabolism (37). Therefore, we analyzed the effects of KD treatment on the key players of theses pathways. Surprisingly, lipogenesis is decreased in the liver of mice fed with KD compared to the chow group, as seen by a lower phosphorylation state of ACC and an important down regulation of SCD1 mRNA expression (Figure 3A). However, no differences in the expression of the mature form of the SREBP-1 was observed. The level of expression of CPT1 was also not affected by KD suggesting that in our experimental conditions, the β-oxidation is not modulated. On the other hand, the mRNA expression of CD36, the main receptor implicated in hepatic lipid uptake was strongly increased in the KD group versus the CHOW group. An increased in PPARα mRNA expression was also observed. Compellingly, this transcription factor is known to be essential in the ketogenesis process (38) (Figure 3A). Our data suggest that the development of hepatic steatosis in mice fed with KD is mainly due to an increase in exogenous lipids uptake and not to *de novo* lipogenesis. Depending on the nature of the dietary fatty acids, an increase in hepatic inflammation can be observed (30). We have shown that KD-fed mice exhibit a slight increase in concentration of hepatic pro-inflammatory cytokine TNF-α compared to the CHOW group, associated with a decrease in the expression of the anti-inflammatory cytokines IFN-β and IL-4 (Figure 3B). Our data suggest that the liver of mice fed for 12 weeks with KD present a pro-inflammatory profile seen in MAFLD (39).

### 3.4 Restauration of metabolic profile after 4 weeks on CHOW diet

**Figure 4:**
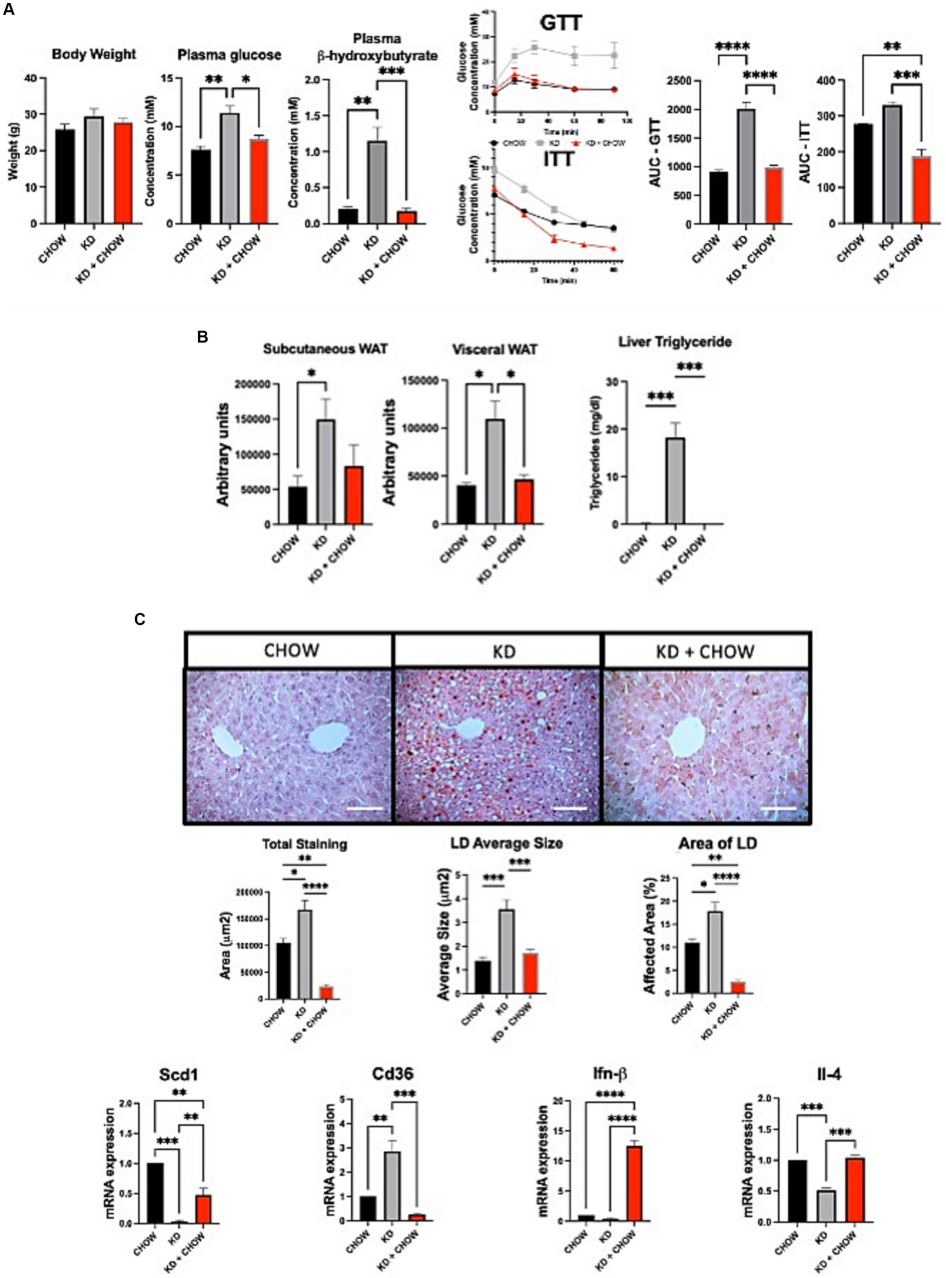
Effect of 4 weeks of CHOW diet after 12 weeks of Ketogenic Diet. **(A)** Evaluation of body weight, plasma β-hydroxybutyrate and fasting glucose, GTT was measured at week 3 and ITT was measured at week 4 after the switch of the diet. AUC represents the calculated area under the curve. **(B)** Representative images of WAT distribution. Red arrows indicate subcutaneous WAT while blue arrows show visceral WAT. Scale Bar = 20 mm (Pictures in Supplementary Data). Quantification was performed using the Particle Analysis function of ImageJ. Liver triglyceride levels. **(C)** Representative images of RedOil and eosin∕hematoxyl staining of hepatic tissues sections. Scale bar = 70 μm. Quantification of total, average size area and percentage area of lipid droplets in liver sections were performed using ImageJ. mRNA expression of Scd1, Cd36, Ifn-β, and Il-4 are presented. Hprt1 were used as control. Data are shown as mean +/− SEM. *n* = 3. Student’s *t*-test: *p-value <0.05, **p-value <0.01, ***p-value <0.001, and ****p-value <0.0001.

Despite presenting some deleterious metabolic features, mice fed with KD for 12 weeks do not appear to develop insulin resistance. Therefore, we evaluated the effect of switching the mice for 4 weeks on a regular CHOW diet after 12 weeks of KD, and if this change could restore a healthier metabolic phenotype. As expected, after 4 weeks on chow diet, the circulating level of ketone bodies was substantially decreased, restoring the levels observed in mice not fed with KD. As seen by the GTT, mice were no longer glucose intolerant. Similar observation was made when we performed ITT (Figure 4A). Moreover, mice have shown a better response of insulin tolerance test compared to the control group (Figure 4A). Switching mice from the KD to CHOW diet for 4 weeks showed that the visceral adipose tissue mass is strongly decreased reaching a similar level to the one observed in CHOW-fed mice. Despite being decreased, the subcutaneous fat mass is not statistically different than the one measured in mice fed only with KD (Figure 4B). The change in diet also totally resolved the hepatic steatosis, TG accumulation and restored a normal inflammatory profile (Figures 4B,C). These results suggest that the deleterious effect of KD on metabolic profile can be rapidly restored by switching the animals to a regular diet for only 4 weeks.

## 4 Discussion

As of today, there is still controversy surrounding the KD diet, particularly regarding its effects on glucose and insulin sensitivities, on weight loss and on MS development. In the present study, we evaluated the effect of 12 weeks of KD on fat mass distribution, glucose and insulin tolerance, hepatic steatosis, lipid metabolism and inflammatory profile in mice. Furthermore, we analyzed the same metabolic features after switching mice from a 12 weeks KD to chow diet for an additional 4 weeks.

As expected, mice fed with KD for 12 weeks in total absence of sugars showed an increase in the plasma concentration of ketone bodies, notably β-hydroxybutyrate (βOHB), a marker of ketosis (7). In response to exogenous fatty acid overload, PPARα is activated inducing the expression of several of its targeted genes involved in ketogenesis, such as CPT1, hydroxymethylglutaryl-CoA synthase 2 (HMGCS 2) or uncoupled protein 2 (UCP2) (40). In our study, exogenous fatty acid overload is also induced by our KD. Additionally, we observed an increase in the expression of PPARα but also of one of its target gene CPT1 enabling the mice to create ketone bodies (40).

As showed in other studies using our KD in absence of any sugar (21, 22), an elevation of fasting plasma glucose and insulin levels was observed. This suggests a development of insulin resistance (41). However, performing GTT and ITT, we showed that our KD-mice are glucose intolerant but remained insulin sensitive. Glucose intolerance is characterized by elevated fasting glucose (IFG) and impaired glucose management upon glucose challenge, also name impaired glucose tolerance (IGT) (42). Patients with IFG and/or IGT are often defined as “Prediabetic” (43). Glucose intolerance represents the first step in the development of insulin resistance as observed in mice fed, for 12 weeks, with an obesogenic diet (60% lard, 25% carbohydrates) (44). Surprisingly, in another study, severe insulin resistance has been observed following exposure to a low-sugar diet (90% fat and 0.3% carbohydrate) within only 3 days (21). In another study, authors showed that 12 weeks of KD (79.1% fat and 3.8% carbohydrate) induce insulin resistance associated with impaired hepatic insulin signaling (22). In contrast, humans’ studies showed that KD (with less than 5% carbohydrates) improve glucose and insulin sensitivities suggesting differences in response to dietary sugars between mice and human (23–25). Moreover, ketone bodies are also known to reduce circulating glucose and insulin levels, thus improving insulin sensitivity (45). Our results showed that 12 weeks of KD also have deleterious effects, although with less severity than the effects observed in other mice studies (21, 22). This can be explained by the total absence of carbohydrate in our experimental conditions suggesting that even a low level of sugar in the diet can induce insulin resistance at least in mice.

In an *ad libitum* context, our KD-fed mice showed an accumulation of both subcutaneous and visceral white adipose tissues, as well as hepatic TG accumulation. Our diet is composed by 40% saturated fatty acids (SFA), known to increase LDL-cholesterol levels (46). SFA is also known to play a crucial role in the development of low-grade inflammation and metabolic diseases such as NASH (30, 46). Dietary palmitate, the most common SFA (around 30% of fat-part of our KD), is known to increase the level of the plasmatic pro-inflammatory cytokine TNF-α and to decrease the levels of hepatic anti-inflammatory cytokines IFN-β and IL-4, as seen in our study (46, 47). IFNβ may play multiple roles as well pro-and anti-inflammatory (48). However, a study performed mice fed with a high fat diet, showed that IFNβ overexpression has been observed to attenuate adipose tissue inflammation, adipose tissue expansion and body weight gain. The authors argue that IFNβ expression helps the high fat diet-fed mice to attenuate production of pro-inflammatory cytokines (such as Tnf-α, Il-1β, and Il-6). In addition, in this study, the authors also showed that IFNβ improves insulin sensitivity and glucose homeostasis (49). As expected, the presence of high concentration of palmitate in our KD, considerably activated the hepatic expression of CD36. High expression of this fatty acid translocase in liver is strongly associated with MAFLD development and elevated hepatic intracellular oxidative stress (46, 50). CD36 also participates in the inflammatory response via the activation of the c-Jun N-terminal kinase pathway (51). *De novo* lipogenesis is also increased during the development of MAFLD, notably through the activation of the transcription factor SREPB-1 (52). In our study, we did not observe induction in the expression of the mature form of SREBP1 (mSREBP1c). On the contrary, an abolition of SCD1 expression and a strong inhibition of ACC phosphorylation was observed. This suggests that 12 weeks of treatment with KD does not induce hepatic lipogenesis and may inhibit it. This could be partially explained by the total absence of carbohydrates in our KD, since hepatocytes derive their acetyl-CoA, the main substrate for hepatic lipogenesis, essentially from carbohydrate catabolism (53). Moreover, a correlation between low SCD1 expression and high ketone body level has been observed in Nuclear Factor erythroid 2-Related Factor 2-KnockOut (Nrf2-KO) mice. The transcription factor Nrf2 activate SCD1 and SREBP1 in response to a diet-induced obesity (54). Interestingly, Nrf2-KO mice develop ketosis. Nrf2 is a transcription factor targeting genes involved in lipid uptake (such as CD36) and lipogenesis (such as SCD1 and SREBP) and its deletion increase the expression of genes involved in lipid oxidation (such as PPARα and CPT1) (54). Ketone bodies production and VLDL secretion is observed in Nrf2-KO mice (55). However, the authors have not identified the molecular mechanisms underlying the link between SCD1 and ketone bodies production (55). In our study, we observed a strong decrease in SCD1 expression without any effect on mSREBP1c expression suggesting a mechanism independent of SREBP-1c that need to be identified. Medium-chain triglycerides (MCT) are also known for their ability to be rapidly converted into ketone bodies. In mice fed an MCT-enriched diet for a year, glucose homeostasis is altered an associated with decreased hepatic SCD1 expression by an unidentified mechanism (56, 57). Taken together, our data suggests that the development of hepatic steatosis observed in our KD-fed mice for 12 weeks is probably due to an increase in SFA uptake and not to an activation of *de novo* lipogenesis.

It is well known that inflammation is involved in the progression from MAFLD to NASH (58). As predicted, our KD-fed mice showed an increase in the expression of the pro-inflammatory marker TNF-α associated with a decrease in the expressions of the anti-inflammatory markers IL-4 and IFN-β. Several studies performed on mice, both wild-type and mice with T2D, demonstrate the harmful impact of KDs in promoting inflammation and participating in the development of hepatic steatosis, notably due to the large quantity of SFA present in the diets from lard (59). On the other hand, several studies in humans show opposite effects. In obese subjects with hepatic steatosis, 14 days of KD [76% fat (mix of lard, butter, and corn oil), 4% carbohydrate] improved the inflammatory profile by reducing the cytokines TNF-α and IL-6 (59). Moreover, induction of ketogenesis or treatment with βOHB attenuates TNF-α-induced apoptosis in human intestinal cell lines, demonstrating a protective effect of ketone body production (60). However, it is well known that high dietary SFA, as in our experimental KD, play an important role in the development of chronic inflammation and is correlated with increased secretion of TNF-α (30) and a decrease in anti-inflammatory cytokines (61–64).

Apart from these negative effects, KDs also offer interesting possibilities. In the second phase of our research, our mice fed KD for 12 weeks were switched to a chow diet. Within only 4 weeks, the mice showed a decrease in visceral adipose tissue mass and an improvement in the hepatic lipid storage compared to the KD group. As expected, the ketosis disappears but, interestingly, most of the metabolic deleterious effects are reversed. The mice showed an improvement in insulin sensitivity, exceeding the results obtained in the control group, fed with chow diet only. Our results showed that even if the zero-sugar KD is used in these experiments, some deleterious effects (mild compared to those observed in other previous studies), this can be reversed by a return to a more normal diet.

Several recent studies performed in both humans and rodents fed intermittent-fasting and/or ketogenic diet also demonstrated an improvement in weight loss, glucose tolerance, insulin sensitivity, chronic inflammation as well as a better MS score (65–67). In another study, mice were fed with an obesogenic (60% lard, 20% carbohydrate) diet for 8 weeks, followed by 4 weeks on either a KD diet (90% cocoa butter fat containing 60% of SFA) or a chow diet (68). Interestingly and in agreement with our observations, the KD diet reversed the development of hepatic steatosis induced by the obesogenic diet, though, to a lower degree than with what is observed with a standard diet (68). The KD diet may present reversible mild deleterious effects in the development of MAFLD for a healthy individual exclusively fed on KD. KD, however, has therapeutical beneficial effects to alleviate the deleterious effects of an obesogenic diet (60% fat, 20% carbohydrates) (69). In fact, switching mice from the obesogenic diet to KD for 8 weeks normalized the glycemic index and significantly reduced the body fat (69).

In conclusion, a KD, in absence of any sugar, can be beneficial for the treatment of diseases such as MAFLD and NASH but also can be used for periodic treatments of seizures, as conveyed in our study. The absence of sugar distinctly decreases the impact on insulin resistance allowing a restauration of healthier metabolic profiles after only 4 weeks on a regular diet. Despite the benefit effects of KD, precautions need to be taken concerning the nature of fats present in the diet. Alternative strategies must be evaluated such as the replacement of SFA by either unsaturated fatty acids or MCT to avoid development of MAFLD and systemic inflammation.

## Data availability statement

The raw data supporting the conclusions of this article will be made available by the authors, without undue reservation.

## Ethics statement

The animal study was approved by Comité institutionnel de protection des animaux (CIPA) de l’UQAM. The study was conducted in accordance with the local legislation and institutional requirements.

## Author contributions

GR: Conceptualization, Data curation, Methodology, Writing – original draft, Writing – review & editing. AC: Methodology. CM: Conceptualization, Funding acquisition, Investigation, Resources, Supervision, Validation, Writing – original draft, Writing – review & editing.
