# Supplementary figures and images for: Exploring the impacts of ketogenic diet on reversible hepatic steatosis: initial analysis in male mice
Source: Front Nutr. 2024 Mar 21;11:1290540. doi: 10.3389/fnut.2024.1290540 (PMC10991688; doi:10.3389/fnut.2024.1290540)

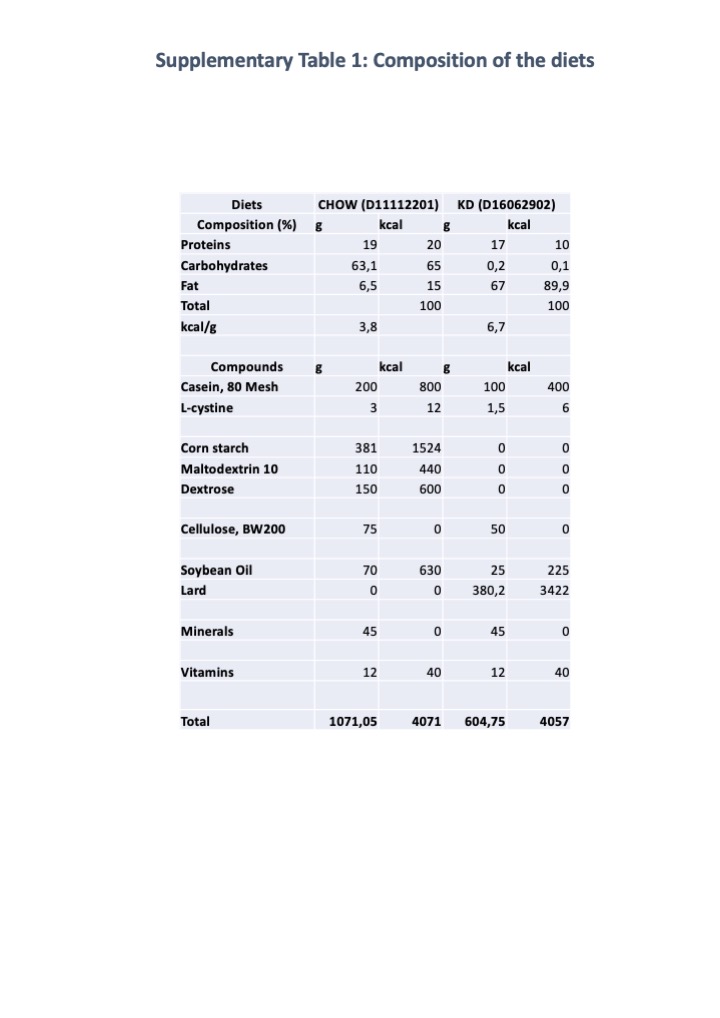

Supplement: Supplementary file 1 [file Image_1.JPEG]
